# Levetiracetam improves disinhibitory behavior in nonconvulsive status epilepticus

**DOI:** 10.1186/s12991-014-0032-0

**Published:** 2014-10-14

**Authors:** Kazuhiko Yamamuro, Hiroki Yoshino, Kentaro Tamura, Toyosaku Ota, Toshifumi Kishimoto

**Affiliations:** Department of Psychiatry, Nara Medical University, 840 Shijo-cho, Kashihara, 634-8522 Nara Japan; Department of Neurosurgery, Nara Medical University, 840 Shijo-cho, Kashihara, 634-8522 Nara Japan

**Keywords:** Disinhibitory behavior, Levetiracetam, Nonconvulsive status epilepticus, Psychiatric disorder, Infectious encephalitis

## Abstract

**Background:**

Nonconvulsive status epilepticus (NCSE) is a severe medical condition and heterogeneous disorder defined by different seizure types and diverse etiologies. NCSE occurs commonly in the elderly and is potentially misdiagnosed as a psychiatric disorder. Current treatment options for NCSE are still unsatisfactory.

**Case presentation:**

We report a case of NCSE in a 55-year-old epileptic male patient with a history of infectious encephalitis, disinhibitory behavior, and a suspected diagnosis of frontotemporal dementia. Add-on levetiracetam (LEV) to carbamazepine treatment improved clinical manifestations and abnormal electroencephalographic discharge.

**Conclusion:**

With disinhibitory behavior in the elderly, the possibility of NCSE should be considered. Moreover, LEV may be an effective and well-tolerated pharmacotherapy for elderly NCSE patients.

## Introduction

Nonconvulsive status epilepticus (NCSE) is common and often manifests as altered consciousness accompanied by subtle motor twitches or ambiguous behavior changes [1], although there are subtypes of NCSE without altered consciousness. Diagnosis is based on electroencephalographic (EEG) recordings and is defined by continuous or repeated EEG epileptic discharges beyond 10 min [2]; and continuous EEG-video monitoring is preferred to increase detection rate of NCSE [3]. Without evidence of continuous EEG epileptiform activities, NCSE without altered consciousness is easily misdiagnosed, leading to delays in treatment or loss of opportunity for proper treatment [4].

Levetiracetam (LEV) is a newer antiseizure drug (ASD), with an approved oral formulation that can be administered at doses effective in controlling seizures [5]. LEV is known to be well-tolerated, even in the elderly [6].

This report focuses on the use of LEV as an add-on therapy in the treatment of NCSE in an epileptic patient with disinhibitory behavior, as our survey of the literature did not identify any current report related to LEV used in this context. We found add-on LEV to carbamazepine (CBZ) treatment reversed clinical and EEG manifestations. Moreover, we highlight the importance of differential NCSE diagnosis in elderly patients with abnormal behavior, such as disinhibition, and the high tolerability and efficacy of LEV about elderly NCSE patients.

## Case report

**Figure 1:**
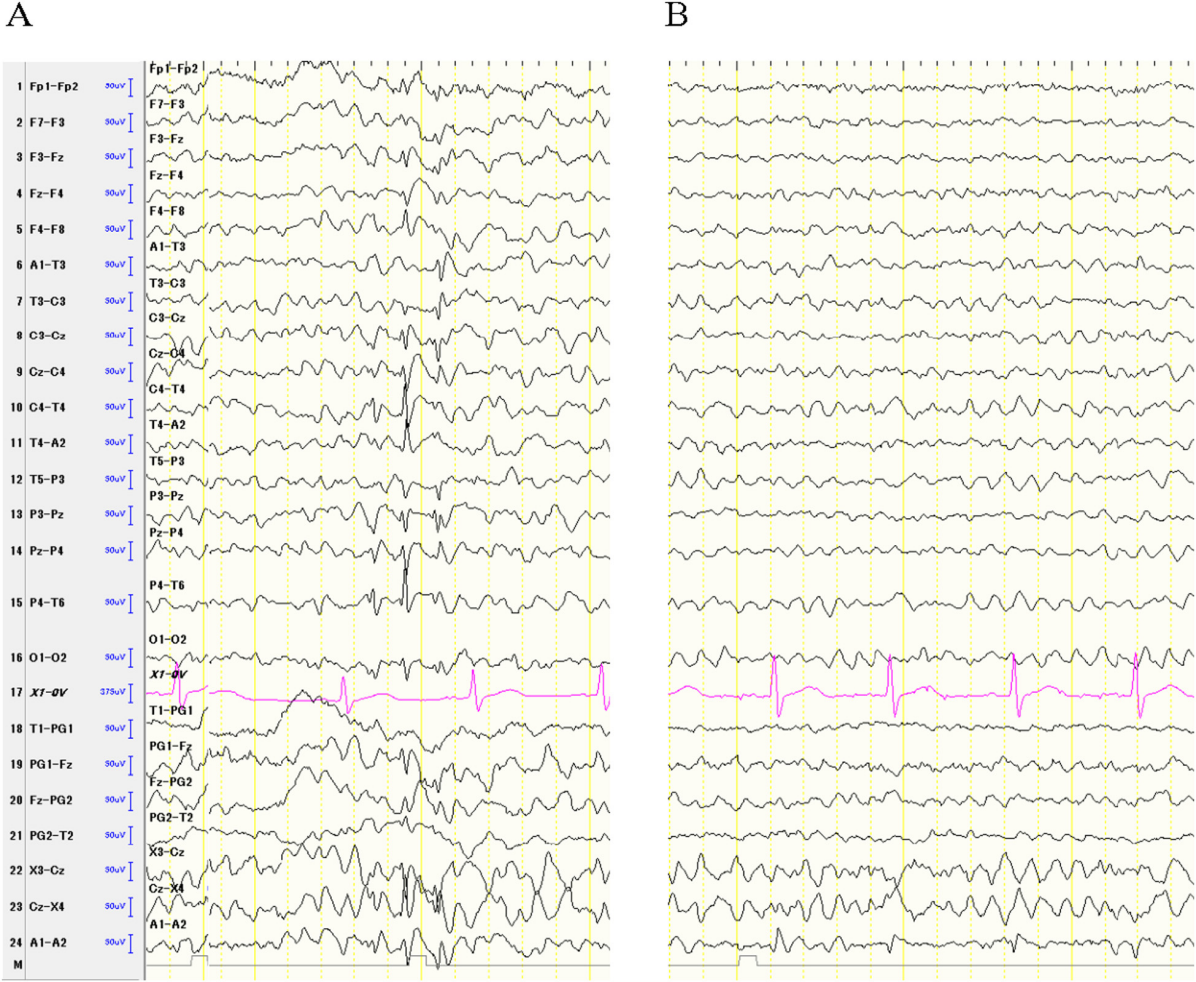
**EEG recordings from the patient. (A)** Persistent ictal discharges of 5 Hz in the right frontal region were observed in an initial EEG. **(B)** A follow-up EEG showed suppressed rhythms in the right frontal region after add-on LEV treatment.

A 47-year-old man was admitted to the hospital with headache and fever and diagnosed with infectious encephalitis (virus not determined/bacteria not detected). He has suffered from tonic-clonic seizures since 50 years of age, with a seizure frequency of one per year, and consequently been administered with CBZ (600 mg/day). At 55 years of age, he suddenly started to show socially inappropriate behavior, often disappearing during work, and taking pictures of unknown woman on the train, culminating in a police warning. To treat his disinhibitory behavior, he was referred to our hospital. His consciousness was normal and his vital signs are stable. There were no focal neurological signs except blepharospasm and there was no obvious convulsion. Admission laboratory work was normal except for elevated γ-GTP (77 U/l). Blood CBZ levels were in the normal range (4.3 mcg/ml). First, we suspected a psychiatric disorder with disinhibition, specifically frontotemporal lobar degeneration. However, we did not observe any abnormal findings in the magnetic resonance imaging and single photon emission computed tomography, whereas EEG examination identified epileptiform discharges with components resembling spikes of ‘spike and slow wave’ complexes, and which occurred at a frequency of 5 Hz in the right frontal region (Figure 1A). Based on a diagnosis of NCSE, he began 1,000 mg/day LEV, which was increased to 2,000 mg/day the following month. Two months later, his disinhibitory behavior and abnormal EEG discharge disappeared, with few side effects (Figure 1B).

## Discussion

To the best of our knowledge, the case presented here is the first report showing the therapeutic effect of add-on LEV for treatment of NCSE with disinhibitory behavior.

Previously, complex partial status epilepticus (CPSE) was thought to be rare [7]; however, subsequent studies have shown CPSE amounts to 16%–43% of all status epilepticus cases [8,9]. CPSE is characterized by clouding of consciousness and is a heterogeneous condition that may be related to ictal disorganization of various temporal or extratemporal epileptogenic networks, mainly involving frontal lesions [10,11]. NCSE of frontal origin occurs frequently without overt confusion [12,13]. Thomas et al. [13] reported that NCSE of frontal origin can be identified by two types. First, type 1 is characterized by mood and behavior, either a hypomanic state related to right frontal focus with affective disinhibition and increased verbal fluency or conversely, a state of emotional indifference related to left frontal focus with diminished facial expression, reduced verbal fluency, and decreased emotion and spontaneous activity, without clear alteration of consciousness, and most patients are able to recall the episode. EEG patterns consist of unilateral frontal ictal activity. Second, type 2 is characterized by temporospatial disorientation and evident behavioral disorder with a confusional state. EEG patterns consist of bilateral frontal ictal activity. Based on these findings, we classified our present case as type 1 NCSE with right frontal lesion.

LEV’s mechanism of action as an ASD involves binding to a synaptic vesicle glycoprotein, SV2A [14], inhibition of presynaptic calcium channels [15], reducing excitatory neurotransmitter release [16], and thereby acting as a neuromodulator. Although LEV requires cautious use for patients with frontal lesions and might bring substantial fatigue and drowsiness for elderly patients [17,18], LEV is relatively well-tolerated and has few side effects [6,19]. Furthermore, previous reports have demonstrated the efficacy of LEV as a co-medication in treatment of status epilepticus and refractory status epilepticus, including patients with NCSE [20,21]. LEV may also serve as an advantageous pharmacotherapy for the treatment of elderly NCSE with disinhibitory behavior.

## Conclusions

NCSE is a heterogeneous disorder including a number of subtypes with varied electroclinical feature manifestations. NCSE should always be considered with the sudden appearance of behavioral and/or cognitive changes in elderly patients, especially those with a past history of organic brain disease. As LEV is effective and well-tolerated, even in the elderly, it may be a better option with fewer side effects, to treat elderly NCSE patients with abnormal behavior.

## Consent

Written informed consent was obtained from the patient for publication of this case report.

## Abbreviations

NCSE: nonconvulsive status epilepticus
LEV: levetiracetam
EEG: electroencephalographic
CBZ: carbamazepine
CPSE: complex partial status epilepticus
ASD: antiseizure drug
